# Multilevel Approach of a 1-Year Program of Dietary and Exercise Interventions on Bone Mineral Content and Density in Metabolic Syndrome – the RESOLVE Randomized Controlled Trial

**DOI:** 10.1371/journal.pone.0136491

**Published:** 2015-09-16

**Authors:** Daniel Courteix, João Valente-dos-Santos, Béatrice Ferry, Gérard Lac, Bruno Lesourd, Robert Chapier, Geraldine Naughton, Geoffroy Marceau, Manuel João Coelho-e-Silva, Agnès Vinet, Guillaume Walther, Philippe Obert, Frédéric Dutheil

**Affiliations:** 1 Clermont Auvergne University, Blaise Pascal University, Laboratory of Metabolic Adaptations to Exercise in Physiological and Pathological conditions (AME2P, EA3533), Clermont-Ferrand, France; 2 Australian Catholic University, Faculty of Health, School of Exercise Science, Melbourne, Victoria, Australia; 3 Research Centre in Human Nutrition (CRNH) Auvergne, Clermont-Ferrand, France; 4 Lusófona University of Humanities and Technologies, Faculty of Physical Education and Sport, Lisbon, Portugal; 5 University of Coimbra, Faculty of Sport Sciences and Physical Education, Research Unit for Sport and Physical Activity (CIDAF), Coimbra, Portugal; 6 Auvergne University, Faculty of Medicine, Geriatric, Clermont-Ferrand, France; 7 University Hospital of Clermont-Ferrand (CHU), Biochemistry, Clermont-Ferrand, France; 8 Laboratory of Pharm-Ecology Cardiovascular (EA4278), University of Avignon, Avignon, France; 9 University Hospital of Clermont-Ferrand (CHU), Preventive and Occupational Medicine, Clermont-Ferrand, France; Garvan Institute of Medical Research, AUSTRALIA

## Abstract

**Background:**

Weight loss is a public health concern in obesity-related diseases such as metabolic syndrome (MetS). However, restrictive diets might induce bone loss. The nature of exercise and whether exercise with weight loss programs can protect against potential bone mass deficits remains unclear. Moreover, compliance is essential in intervention programs. Thus, we aimed to investigate the effects that modality and exercise compliance have on bone mineral content (BMC) and density (BMD).

**Methods:**

We investigated 90 individuals with MetS who were recruited for the 1-year RESOLVE trial. Community-dwelling seniors with MetS were randomly assigned into three different modalities of exercise (intensive resistance, intensive endurance, moderate mixed) combined with a restrictive diet. They were compared to 44 healthy controls who did not undergo the intervention.

**Results:**

This intensive lifestyle intervention (15–20 hours of training/week + restrictive diet) resulted in weight loss, body composition changes and health improvements. Baseline BMC and BMD for total body, lumbar spine and femoral neck did not differ between MetS groups and between MetS and controls. Despite changes over time, BMC or BMD did not differ between the three modalities of exercise and when compared with the controls. However, independent of exercise modality, compliant participants increased their BMC and BMD compared with their less compliant peers. Decreases in total body lean mass and negative energy balance significantly and independently contributed to decreases in lumbar spine BMC.

**Conclusion:**

After the one year intervention, differences relating to exercise modalities were not evident. However, compliance with an intensive exercise program resulted in a significantly higher bone mass during energy restriction than non-compliance. Exercise is therefore beneficial to bone in the context of a weight loss program.

**Trial Registration:**

ClinicalTrials.gov NCT00917917

## Introduction

Most metabolic diseases are linked to excess weight or obesity [1]. All appropriate treatments involving weight loss therapy currently include a restrictive diet. However, restrictive diet may induce bone loss [2,3]. Bone loss accompanying body weight reduction remains debatable [4,5,6,7]. Variable results depend on age, gender and lifestyle: physical activity [8], and protein [4] or calcium intake [9] of individuals striving for weight loss. There is a growing consensus that the risk of bone loss [10] is independent of the severity of dieting, as well as the amount of body weight lost [11,12].

Discussions of the mechanisms for bone loss in weight loss programs have included the potential for a reduction of mechanical loading combined with a lack of calcium and energy intake [2], altered local bone cells interaction [13] and/or reduced expression of transcription factors influencing osteoblastogenesis [14]. Moreover, energy restriction can alter the plasma concentration of hormones involved in bone metabolism and increase the bone turnover in obese woman [15].

The specificity of metabolic syndrome (MetS) [16] tends to target obesity at an abdominal location [16]. Abdominal fat tissue produces numerous adipokines linked with inflammation [17]. Therefore, during lifestyle intervention, inflammatory effects from abdominal fat tissue may occur despite body weight reduction [18].

The benefits of weight-bearing physical activity on bone mineral content (BMC) and density (BMD) are well established [19], and as such, exercise training may also attenuate the decrease in BMD during a weight loss [7,20]. Nonetheless, there are conflicting reports on whether exercise accompanying a weight loss program is able to protect against a potential bone loss. Both diet-induced and exercise-induced weight loss have been linked to bone deficits [21]. Indeed, the bones’ response to weight loss combined with exercise could be specific to weight-bearing sites. Moreover, compliance with physical activity and dietary guidelines has been reported to be essential in the success of lifestyle interventions [22].

The RESOLVE trial (REverse metabolic SyndrOme by Lifestyle and Various Exercises) is an interventional study that assessed the change in elderly participants with MetS after one year [23]. It aimed to compare the broad health-related effects of three different modalities of exercise (intensive resistance, intensive endurance, and moderate mixed) combined with a restrictive diet. This intensive lifestyle intervention resulted in weight loss, body composition changes and improvement of participants’ health [23,24]. To understand more about associated bone changes we also assessed BMC and BMD at each step of the intervention.

Multilevel statistical models are appropriate to deal with repeated measurements. Therefore, the purpose of the present study was to determine the effects of weight loss and the associated characteristics of MetS on bone metabolism, taking into account a physical activity program implementing three different modalities of exercise. If physical activity was capable of compensating for the bone loss induced by the restrictive diet, we further investigated whether any specific modality of exercise induced a better result than the others. A second purpose was to assess the effects of compliance to diet and exercise by measuring the observance of participants during a 3-week residential program followed by 11 months of at-home intervention.

## Methods

### Participants

Volunteers were recruited via advertisements. They gave their written informed consent. The study was reviewed and approved by the human research ethics committee from the University Hospital of St Etienne, France. To be eligible, participants had to be: aged between 50 and 70 years, symptomatic of MetS [16], leading a sedentary lifestyle, stable in body weight and medical treatment over the previous 6 months, post-menopausal for women, without hepatic, renal, or psychiatric diseases, nor cardiovascular or endocrine diseases except those defining MetS, devoid of medications altering bone tissue properties, not undertaking restrictive dieting in the previous year, and able to complete a normal maximal exercise tolerance test (VO_2max_).

For baseline references, data were obtained from an age-matched control group of healthy participants who did not undergo any intervention. Criteria for inclusion as a healthy control included being without any of the defined criteria of MetS, chronic disease, and routine medication. They also had to report a stable lifestyle over the previous 12 months, and at the time of testing were completing less than three hours of organized physical activity per week.

### Primary outcome

The primary outcome was the change in BMC and BMD for the whole body, lumbar spine and non-dominant hip. BMC and BMD were measured by dual-energy X-ray absorptiometry scans (DXA, Hologic QDR 4500 series; Waltham, USA). The in-vivo coefficient of variation (CV) was 0.5%.

### Secondary outcomes

#### Anthropometry

Body height was measured with a standard stadiometer (Holtain, Ltd., Crymych, UK). Fat mass and lean body mass were measured by DXA, with respective in vivo CV of 4.2 and 0.4%. Central fat, a surrogate of visceral fat, was assessed from DXA scans, as described by Kamel et al [25]. We determined a CV of 1.6% in the central fat measurements.

#### Biochemistry

Fasting blood samples were drawn between 7.00 and 7.30 a.m., aliquoted and stored at -80°C until analysis. Basic biological assays were performed in the biochemistry laboratory of the University Hospital of Clermont-Ferrand, France. Insulin, pro-inflammatory cytokines (TNF-alpha, IL-1 and IL-6), the adipokines leptin and adiponectin, and PAI-1 were assayed by ELISA using commercial kits (Millipore, Billerica, MA, USA). Sensitivity, intra- and interassay coefficients of variation were respectively 3.0 ng/ml, 2.6%, 7.2% for insulin; 0.7 pg/ml, 6.0% and 9.0%, for TNF-alpha; 1.3 pg/ml, 9.0% and 9.0% for IL-1; 1.3 pg/ml, 7.0% and 10.0% for IL-6; 0.16 ng/ml, 5.1% and 7.4% for leptine; 0.78 ng/ml, 0.9% and 2.4% for adiponectine; 1.3 pg/ml, 6.6% and 10% for active PAI-1 and total PAI-1. Insulin resistance was estimated by calculating of the homeostasis model assessment-insulin resistance (HOMA-IR) index (fasting plasma glucose x fasting plasma insulin)/22.5. Bone metabolism markers included the serum concentration of osteocalcin, which was assayed by ELISA (N-MID Osteocalcin ELISA, Nordic Bioscience Diagnostics A/S, Denmark). Intra- and interassay CVs were 2.6% and 4.7%, respectively, with a sensitivity of 0.5 ng/ml. Other bone metabolism markers were Procollagen type I N-terminal propeptide (PINP) and type I-C telopeptide breakdown products (CTX) which were assayed using Cobas 6000 (Roche Diagnostic, Mannheim, Germany) with intra and inter-assay CVs lower than 7%.

#### Exercise and nutrition

Three-day self-report questionnaires on food intake and physical activity allowed the calculation of daily energy intake and daily energy expenditure, as well as daily calcium and protein intake. Routine medications were recorded and all participants were advised to maintain their baseline medications.

### Time of measurements

Outcomes were measured at baseline (Day 0), 21 days (D21), 3 months (D90), 6 months (D180) and 12 months (D360), with the exception of bone biomarkers which were measured at D0, D21, and D180. Self-reported questionnaires on food intake and physical activity were completed each month.

### Randomization

Stratified computer-generated randomization (combined with permuted blocks to produce balanced allocation within each stratum) was used to account for sex, age, and body mass index, then participants were assigned to one of the following groups:

- *Re*: high-Resistance-moderate-endurance, 10 repetitions performed at 70% of one maximal repetition for resistance and 30% of VO_2_-peak for endurance training,
- *rE*: moderate-resistance (30%)-high-Endurance (70%),
- *re*: moderate-resistance (30%)-moderate-endurance (30%).

Sex was incorporated as a dummy coded variable. Baseline age was divided at the median (≤60.8 years and >60.8 years) and BMI at the tertiles (≤30.6 kg.m^-2^, 30.7 to 36.6 kg.m^-2^, and >36.6 kg.m^-2^) were also applied to stratification. Based on the number and levels of stratification factors, 12 strata were considered with ≤1% risk of a value smaller than 3 participants per stratum [26].

All participants followed the same restrictive diet. Assessors for all outcomes were blinded to the participants’ group assignment. All outcome data remained blinded until the completion of the intervention.

### First stage of intervention: A 3-week residential program

Each day, throughout the residential program, the patients received both standard and personalized balanced meals prescribed by dieticians. Protein accounted for 15 to 20% of the total energy intake (1.2 g.kg^-1^.day^-1^ to maintain protein homeostasis) [27], 30 to 35% were lipids, and carbohydrates comprised the remaining composition of the food groups. Participants’ total daily food intake was calculated to enable them to reach a negative energy balance of ~2090 kJ per day (i.e. 500 kcal per day). Participants were individually coached on daily basis, within the context of their assigned group. An equal amount of time (15–20 h/week) was spent by all groups in endurance (90 min daily) and in resistance (90 min, four days a week). Exercises differed only in intensity, from 30% to 70%. Participants’ heart rates were monitored by Polar S810 with instantaneous recording and storage of heart rate values. Endurance training included aquagym, cycling and walking. Resistance training consisted of 8 exercises with free weights and traditional muscle building equipment. Each exercise was performed for three sets of 10 repetitions.

### Second stage of intervention: A 1-year at-home follow-up

From D21 to D360, the participants were required to complete the same training program without supervision. At D90, D180 and D360, the participants were seen by the dietician and a physical coach for individual adjustments to their program.

A compliance score was determined on the basis of the number of food questionnaires returned (score from 0 to 12 i.e. 12 = 100%) and the number of training sessions undertaken per week (score from 0 to 4, i.e. 4 = 100%). The overall compliance score was the mean of these two scores (nutrition and physical activity). The participants who reached a score of 60% and over were considered as compliant.

### Statistical analysis

The number of participants for each group was based upon the amount of expected central fat loss [23]. A sample of 30 participants per intervention group allowed a statistical power greater than 80% with an α level less than 5%, allowing a dropout rate of 20%.

Descriptive results are presented as means ± standard deviation (SD) unless otherwise noted. The Gaussian distribution for each parameter was assessed by a Shapiro-Wilk test. In case of non-normal distribution, data were log-transformed for analysis. Baseline and follow-up characteristics were cross-sectionally compared between groups using analysis of variance (ANOVA). Alpha levels were set at 0.05 and were adjusted using the Bonferroni technique for multiple comparisons; for example, for 10 comparisons, the α corrected for experiment-wise error rate would be 0.05/10 = 0.005. All these routine analyses were assessed using SPSS software v19.0 (IBM Company, NY, USA).

For the analysis of the change, taking into account the multilevel design of the program [level 1 units (individuals stratified by age, sex and body mass index) within each level 2 unit (individuals of different MetS groups)], hierarchical random effects models (REM) were constructed using a multilevel modeling approach (MLwiN v2.26, Center for Multilevel Modelling, University of Bristol, Bristol, UK) [28]. Analysis models that contained variables measured at different levels of a hierarchy are known as multilevel regression models. The following additive polynomial multilevel model was adopted to describe the changes in BMC and in BMD from study entry:

$${\text{y}}_{ij}\;=\;(\alpha \;+\;\mu_{j})\;+\;(\beta \;+\;v_{j})\;x_{ij}\;+\;({ɀ}_{1ij}\;+\;{ɀ}_{2ij}\;+\;\cdots \;+\;{ɀ}_{nij})\;+\;{ɛ}_{ij}$$

This equation is an example of REM in which level 1 regression coefficients are treated as random variables at level 2. In this example, the number of days from baseline (*x*) is in both the fixed and random parts of the model. This is seen clearly when equation 1 is rearranged into fixed and random parts:

$${\text{y}}_{ij}\;=\;\left(\alpha \;+\;\beta_{j}\;x_{ij}\;\right)+\;\left({ɀ}_{1ij}\;+\;{ɀ}_{2ij}\;+\;\cdots \;+\;{ɀ}_{nij}\right)+\;(\mu_{j}\;+\;v_{j}\;x_{ij}\;+\;{ɛ}_{ij})$$

where *y* is the bone parameter [BMC (g) or BMD (g/cm^2^)] on measurement occasion *i* in the *j*th individual, α is the constant for each *j*th individual, β_*j*_ *x*_*ij*_ is the slope for the bone parameter over time (in these models, the number of days from baseline was centered around day 180, the middle time point of the study) for the *j*th individual; and ɀ_*1*_ to ɀ_*n*_ were the coefficients of explanatory variables (e.g., intervention group, total body lean mass, vitamin D, etc.) at assessment occasion *i* in the *j*th individual [28,29]. These were the fixed parameters in the model.

Both μ_*j*_, *v*_*j*_x_*ij*_ and ε_*ij*_ formed the random parameters in the model. They were assumed to be independent and follow a normal distribution, with means equal to zero and variance σ^2^. ε_*ij*_~*N*[0,var(ε_*ij*_)] was the level 1 residual (within-individual variance) for the *i*th assessment of BMC (g) or BMD (g/cm^2^) in the *j*th individual. Also, *μ*_*j*_~*N*[0,var(*μ*)] was the between-individuals’ intercept variance and *v*_*ij*_ *x*_*ij*_~*N*[0,var(*v*_*ij*_ *x*_*ij*_)] was the between-individuals’ slope variance; thus being used as the level 2 residuals (between subjects) variances for the *j*th individual. The equation *μ*_*j*_×*v*_*ij*_ *x*_*ij*_~*N*[0,var(*μ*_*j*_×*v*_*ij*_ *x*_*ij*_)] explained the intercept-slope covariance relationships among the intercepts and slopes in the model [28,29].

Models were built using a stepwise procedure, i.e. predictor variables (*z* fixed effects) were added one at a time, and likelihood ratio statistics were used to judge the statistical fit of the model [28]. Predictor variables (*z*) were accepted as significant if the estimated mean coefficient was greater than twice the standard error of the estimate. If the retention criterion was not met, the predictor variable was discarded. Power functions for the number of days from baseline were introduced into the linear models to allow for the nonlinearity of changes in bone parameters. Based on analytical [i.e., Pearson’s product moment correlation coefficients (*r*_*y*,*x*_) between bone parameters (*y*) and potential predictors (*x*) at baseline, D21, D90, D180 and D360] and biological assumptions, the following variables were considered in the multilevel models: days from baseline, days from baseline^2^, age at study start, total body lean mass, total body fat mass, central fat mass, energy intake, protein, calcium, vitamin D, CRP, hsCRP and leptin. Dummy variables were created for MetS, sex and compliance groups with *re* participants, female participants and non-compliant participants as the respective reference categories. Neither insulin, HOMA-IR, pro-inflammatory cytokines (TNF-alpha, IL-1 and IL-6), adiponectin, active and total PAI-1, nor bone metabolism markers (osteocalcin, PINP, and CTX) were significantly related with bone parameters at any measurement occasion, thus they were discarded from the multilevel analysis. A tolerance > 0.10 and a variance inflation factor < 10 were set to avoid collinearity between explanatory variables [30]. A total of six independent multilevel REMs were constructed, specifically: whole body BMC, lumbar spine BMC, femoral neck BMC, whole body BMD, lumbar spine BMD, and femoral neck BMD.

## Results

### Participants characteristics at baseline

**Fig 1:**
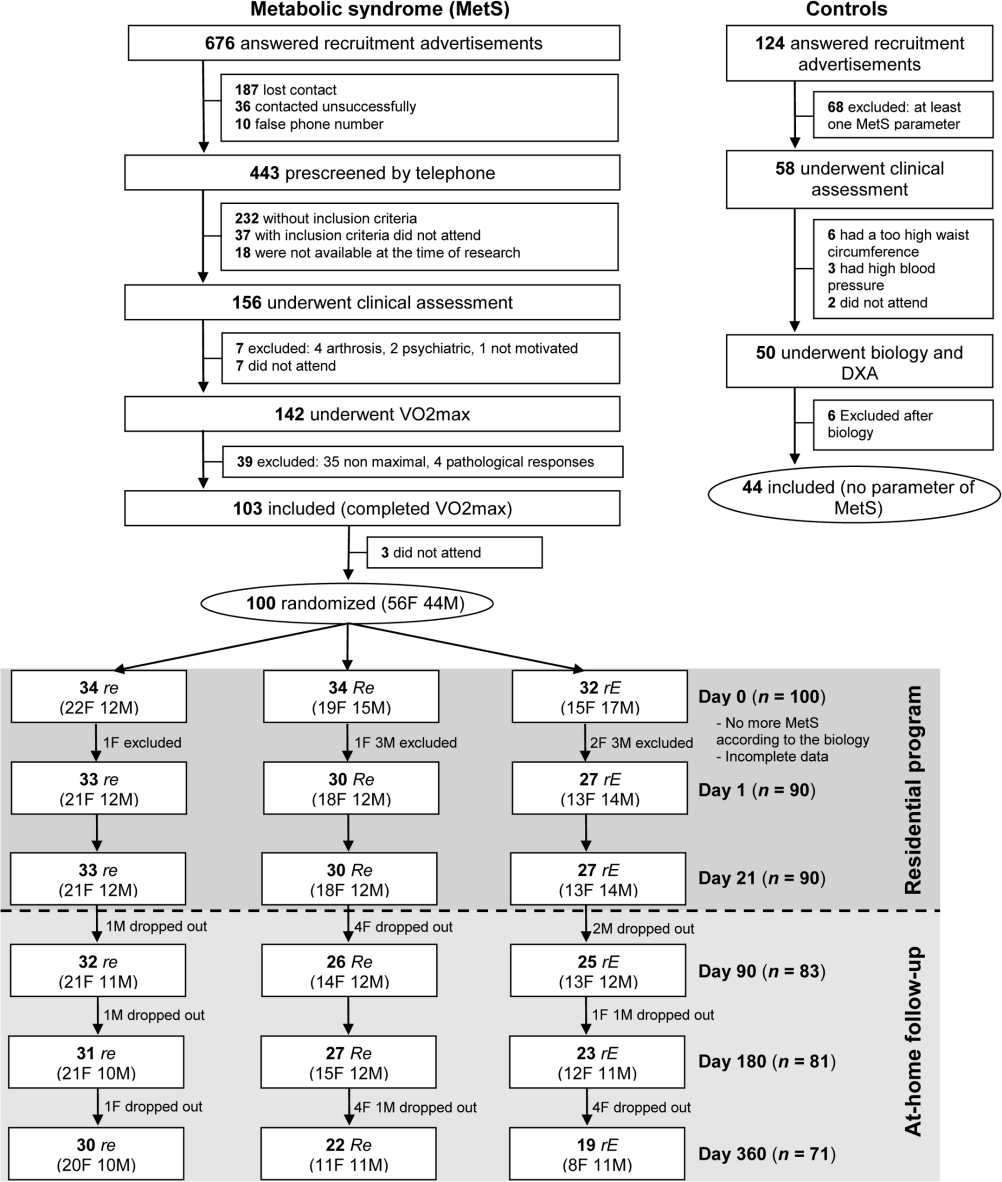
Flow chart of participants. *re*: moderate-resistance-moderate-endurance; *Re*: high-Resistance-moderate-endurance; *rE*: moderate-resistance-high-Endurance.

For the intervention groups, a total of 100 volunteers underwent randomization. Eligible participants were symptomatic of MetS according to the biological tests on D1. Data were retained for analyses only when complete data on primary and secondary outcomes were available. Accordingly, complete data were available for 90 participants at baseline and 71 (79%) completed the whole intervention (Fig 1). No difference in descriptive characteristics, including bones, was found between participants who dropped out of the program and those who completed it. No significant differences were observed in baseline bone parameters between groups (Table 1). However, *rE* were younger and heavier than *Re* and had higher levels of vitamin D than *re* and *Re*. No differences in baseline lumbar spine BMC and in BMD parameters were observed between the sexes either within or between groups.

**Table 1:** Baseline descriptive characteristics of the intervention groups and controls.

	All *i*-groups	*i*-groups			*P*-value	Control	Control *vs*. *i*-groups	
	(*n* = 90)	*re* (*n* = 33)	*Re* (*n* = 30)	*rE* (*n* = 27)	*i*-groups	(*n* = 44)	P-value	*Post-hoc*
Age (years)	60.50 ± 5.19	60.66±5.49	62.18±4.52	58.43±4.97	0.022 ^a^	57.89±4.78	0.001	*C* < *Re*
Sex (F/M–*n*)	52/38	21/12	18/12	13/14	−	21/23	−	−
Height (cm)	164.9 ± 8.6	163.8±8.9	163.5±6.7	167.9±9.7	0.106	170.2±8.0	0.001	*C* > *re*, *Re*
Weight (kg)	91.3 ± 13.0	91.4±12.7	87.1±11.8	95.9±13.4	0.037 ^a^	69.2±12.1	<0.001	*C* < *re*, *Re*, *rE*
Total body lean mass (kg)	60.3 ± 10.9	58.8±11.2	58.7±10.1	64.0±11.1	0.117	50.8±11.0	<0.001	*C* < *re*, *Re*, *rE*
Total body fat mass (kg)	31.0 ± 7.8	32.5±7.6	28.4±7.7	31.9±7.7	0.080	16.1±3.9	<0.001	*C* < *re*, *Re*, *rE*
Central fat mass (kg)	3.1 ± 0.7	3.1±0.7	3.0±0.7	3.1±0.7	0.638	1.2±0.6	<0.001	*C* < *re*, *Re*, *rE*
Energy intake (KJ.d^-1^)	7590.9 ± 2105.4	7095.2±1643.0	7797.4±1869.9	7967.4±2728.1	0.227	−	−	−
Protein (g.d^-1^)	79.7 ± 21.0	73.7±16.6	80.3±18.3	86.4±26.4	0.063	−	−	−
Calcium (mg.d^-1^)	714.2 ± 296.4	661.4±193.8	781.4±360.4	704.0±317.5	0.272	−	−	−
Vitamin D (ng.ml^-1^)	16.93 ± 8.05	14.72±5.41	15.76±5.03	20.94±11.54	0.006 ^a^ ^,^ ^b^	21.24±6.98	<0.001	*C* > *re*, *Re*
C-reactive protein (mg.l^-1^)	5.25 ± 3.42	6.06±4.07	4.54±2.83	5.04±3.02	0.197	3.06±0.74	<0.001	*C* < *re*, *rE*
hs C-reactive protein (mg.l^-1^)	4.57 ± 3.91	5.24±4.57	4.00±3.39	4.40±3.56	0.441	1.25±1.49	<0.001	*C* < *re*, *Re*, *rE*
Leptin (ng.dl^-1^)	3.06 ± 1.57	3.44±1.68	2.74±1.44	2.95±1.53	0.195	1.21±1.08	<0.001	*C* < *re*, *Re*, *rE*
Bone variables								
Total body BMC (g)	2259.67 ± 392.57	2207.40±380.06	2229.05±381.93	2357.57±415.38	0.297	2356.07±459.52	0.291	−
Lumbar spine BMC (g)	67.05 ± 15.45	66.58±16.43	65.88±14.81	68.93±15.32	0.745	69.80±16.36	0.692	−
Femoral neck BMC (g)	4.27 ± 0.72	4.21±0.67	4.22±0.73	4.41±0.77	0.511	4.25±0.94	0.770	−
Total body BMD (g.cm^-2^)	1.13 ± 0.10	1.12±0.09	1.14±0.11	1.15±0.11	0.618	1.16±0.12	0.583	−
Lumbar spine BMD (g.cm^-2^)	1.05 ± 0.17	1.05±0.18	1.04±0.15	1.07±0.16	0.790	1.03±0.17	0.758	−
Femoral neck BMD (g.cm^-2^)	0.81 ± 0.11	0.82±0.11	0.79±0.12	0.83±0.11	0.411	0.78±0.14	0.234	−

Abbreviations: *i-*groups, intervention groups; *re*, moderate-resistance-moderate-endurance; *Re*, high-Resistance-moderate-endurance; *rE*, moderate-resistance-high-Endurance; *C*, control; F, females; M, males; hs, high-sensitivity; BMC, bone mineral content; BMD, bone mineral density.

^a^ *Re* significantly different than *rE* (*P*<0.05).

^b^ *re* significantly different than *rE*. (*P*<0.05). *re* did not significantly differ from *Re*.

The control group (44 participants with no MetS parameters) was matched with the overall MetS group for age and sex. No differences in bone variables between the controls and individuals with MetS were found. However, controls were significantly taller than *re* and *Re*, with less weight, total lean body mass, fat mass, and central fat mass than *re*, *Re* and *rE*. Controls also had higher levels of vitamin D than *re* and *Re* and lower levels of C-reactive protein (CRP), high-sensitivity CRP (hsCRP) and leptin than *re*, *Re* and *rE* (Table 1).

### Cross-sectional MetS-related variation during follow-up

The descriptive characteristics for the *re*, *Re* and *rE* groups from D21 until D360 are presented in Table 2. Cross sectional analyses revealed that at D21 *Re* had significantly lower body mass than *rE* and less total body fat mass and central fat mass than *re*. Also, *re* had lower energy intake than *rE*. At D90, *re* maintained significantly higher levels of total body fat and central fat mass than *Re*. At D180, *re* had significantly lower energy intake, protein, vitamin D and leptin than *rE*. At D360, *re* had higher total body fat mass than *Re*, while consuming less energy than *rE* and less protein than *Re* and *rE* (Table 2).

**Table 2:** Descriptive statistics of anthropometric, body composition, food intake, biochemical data and bone variables for the intervention groups, from day 21 to day 360.

	All *i*-groups	*i*-groups			P-value	*Post-hoc*
	(*n* = 325)	*re* (*n* = 126)	*Re* (*n* = 105)	*rE* (*n* = 94)		
Age (years)						
D21	60.56±5.20	60.72±5.49	62.24±4.52	58.49±4.97	0.022	*Re > rE*
D90	60.78±5.21	61.04±5.53	62.17±4.47	58.76±5.09	0.057	−
D180	61.06±5.02	61.57±5.38	62.33±4.41	58.86±4.68	0.038	*Re > rE*
D360	61.69±4.88	61.82±5.51	63.48±3.81	59.41±4.16	0.026	*Re > rE*
Sex (F/M–*n*)						
D21	52/38	21/12	18/12	13/14	−	−
D90	48/35	21/11	14/12	13/12	−	−
D180	48/33	21/10	15/12	12/11	−	−
D360	39/32	20/10	11/11	8/11	−	−
Compliance (Non-CP/CP–*n*)						
D21	27/63	11/22	7/23	9/18	−	−
D90	24/59	11/21	6/20	7/18	−	−
D180	23/58	11/20	6/21	6/17	−	−
D360	17/54	10/20	5/17	3/16	−	−
Height (cm)						
D21	165.2±8.9	163.9±8.9	164.1±7.6	168.0±9.8	0.143	−
D90	165.1±9.0	163.8±9.1	164.5±8.1	167.3±9.7	0.309	−
D180	164.7±8.6	163.0±8.0	164.5±7.9	167.3±9.8	0.188	−
D360	164.9±8.9	163.3±8.6	164.6±8.7	167.8±9.5	0.228	−
Weight (kg)						
D21	87.9±12.3	88.2±12.1	83.7±11.3	92.0±12.4	0.037	*Re < rE*
D90	84.3±11.9	85.3±12.3	80.6±10.9	86.9±11.7	0.133	−
D180	84.5±12.7	84.9±12.6	81.7±12.4	87.2±13.2	0.311	−
D360	84.2±12.7	85.4±13.1	80.3±11.2	86.8±13.3	0.212	−
Total body lean mass (kg)						
D21	59.5±10.5	57.8±10.7	58.3±9.7	62.9±10.6	0.130	−
D90	58.2±10.5	56.8±11.3	57.6±9.8	60.5±10.0	0.392	−
D180	58.4±10.3	56.6±10.8	58.3±9.9	61.1±10.0	0.285	−
D360	58.2±10.9	56.5±11.4	57.9±10.6	61.4±10.2	0.309	−
Total body fat mass (kg)						
D21	28.3±7.5	30.3±7.4	25.4±7.2	29.1±7.4	0.027	*re* > *Re*
D90	26.1±7.3	28.5±6.8	22.9±7.4	26.4±7.0	0.015	*re* > *Re*
D180	26.0±8.1	28.3±6.8	23.4±8.6	26.1±8.3	0.067	−
D360	25.9±7.7	28.8±7.3	22.4±6.7	25.5±8.0	0.010	*re* > *Re*
Central fat mass (kg)						
D21	2.7±0.6	2.9±0.6	2.5±0.6	2.7±0.6	0.016	*re* > *Re*
D90	2.5±0.6	2.7±0.6	2.2±0.5	2.4±0.6	0.036	*re* > *Re*
D180	2.4±0.7	2.6±0.7	2.2±0.8	2.3±0.7	0.105	−
D360	2.5±0.7	2.7±0.7	2.3±0.7	2.4±0.8	0.066	−
Energy intake (KJ.d^-1^)						
D21	6534.0±511.4	6371.0±572.1	6516.4±512.6	6752.7±340.1	0.014	*re* < *rE*
D90	6704.4±1087.7	6510.0±961.7	6882.0±1257.2	6768.7±1054.6	0.411	−
D180	6673.0±965.5	6330.2±773.5	6831.9±1187.6	6948.6±794.4	0.036	*re* < *rE*
D360	6588.3±931.6	6390.0±821.7	6696.6±1073.7	6775.9±908.3	0.301	−
Protein (g.d^-1^)						
D21	101.6±11.1	99.1±11.7	100.7±11.3	105.5±9.1	0.070	−
D90	84.1±13.4	83.4±14.2	82.3±10.4	86.8±15.1	0.464	−
D180	82.2±14.9	76.2±16.3	81.9±12.4	90.7±11.7	0.001	*re* < *rE*
D360	81.2±10.6	78.8±10.7	79.2±10.5	87.3±8.3	0.011	*re*, *Re* < *rE*
Calcium (mg.d^-1^)						
D21	830.2±103.7	835.1±98.1	830.3±100.4	824.2±116.7	0.922	−
D90	769.1±202.9	766.8±258.2	792.8±159.6	747.5±163.9	0.730	−
D180	811.3±137.3	788.0±140.0	802.8±141.8	852.8±124.3	0.214	−
D360	778.2±129.8	776.2±143.2	785.4±147.4	773.0±84.1	0.950	−
Vitamin D (ng.ml^-1^)						
D21	21.53±17.13	19.29±10.19	18.49 ± 6.21	27.64±27.89	0.083	−
D90	18.92±10.62	18.88±12.20	17.40 ± 5.03	20.56±12.69	0.572	−
D180	19.31±9.33	15.56±4.30	21.52 ± 8.78	21.76±12.94	0.015	*re* < *Re*, *rE*
D360	21.12±6.76	20.07±7.29	20.64 ± 5.86	23.33±6.71	0.242	−
C-reactive protein (mg.l^-1^)						
D21	4.35±3.09	4.86±3.22	3.67±3.11	4.47±2.88	0.308	−
D90	4.93±4.67	5.35±4.12	5.54±6.70	3.74±1.92	0.317	−
D180	4.15±2.32	4.61±2.83	3.85±1.99	3.89±1.84	0.384	−
D360	3.76±1.69	3.91±1.49	3.32±1.72	4.02±1.94	0.346	−
hs C-reactive protein (mg.l^-1^)						
D21	3.17±3.66	3.86±4.15	2.48±3.64	3.10±2.95	0.331	−
D90	3.94±4.92	4.52±4.39	4.19±6.93	2.96±2.50	0.479	−
D180	2.87±2.67	3.19±3.13	2.33±1.98	3.08±2.73	0.431	−
D360	2.51±2.49	2.76±2.05	2.03±2.87	2.67±2.73	0.560	−
Leptin (ng.dl^-1^)						
D21	2.41±1.78	2.69±1.37	2.19±2.05	2.31±1.90	0.513	−
D90	2.14±1.89	2.46±1.51	1.66±1.72	2.24±2.40	0.260	−
D180	2.33±1.89	3.00±1.86	2.14±2.06	1.63±1.46	0.024	*re* > *rE*
D360	1.96±1.34	2.28±1.41	1.81±1.33	1.65±1.20	0.226	−
Bone variables						
Total body BMC (g)						
D21	2267.54±391.68	2211.90±374.90	2234.42±369.07	2372.35±428.52	0.247	−
D90	2234.77±398.57	2196.47±393.54	2221.97±378.53	2297.09±432.77	0.633	−
D180	2227.28±387.18	2166.26±381.54	2208.52±372.50	2331.55±406.92	0.290	−
D360	2248.88±391.91	2181.22±383.08	2236.35±400.50	2370.22±387.97	0.258	−
Lumbar spine BMC (g)						
D21	68.31±16.14	67.34±17.18	67.24±15.34	70.69±16.07	0.662	−
D90	67.95±16.33	68.22±17.30	66.53±15.30	69.09±16.64	0.852	−
D180	67.50±16.38	66.75±17.45	66.41±15.72	69.77±16.16	0.736	−
D360	67.98±16.40	66.14±17.58	67.10±16.24	71.92±14.76	0.469	−
Femoral neck BMC (g)						
D21	4.32±0.77	4.22±0.74	4.28±0.73	4.51±0.85	0.326	−
D90	4.26±0.75	4.25±0.79	4.24±0.69	4.29±0.80	0.972	−
D180	4.27±0.74	4.25±0.78	4.19±0.70	4.40±0.75	0.581	−
D360	4.21±0.71	4.11±0.70	4.18±0.71	4.37±0.71	0.453	−
Total body BMD (g.cm^-2^)						
D21	1.14±0.10	1.12±0.09	1.15±0.09	1.15±0.11	0.437	−
D90	1.14±0.10	1.12±0.09	1.14±0.10	1.15±0.11	0.540	−
D180	1.13±0.10	1.12±0.10	1.13±0.10	1.15±0.10	0.437	−
D360	1.14±0.10	1.12±0.10	1.14±0.11	1.17±0.11	0.412	−
Lumbar spine BMD (g.cm^-2^)						
D21	1.06±0.17	1.05±0.19	1.05±0.15	1.08±0.17	0.729	−
D90	1.05±0.17	1.06±0.19	1.03±0.14	1.07±0.18	0.659	−
D180	1.05±0.17	1.05±0.20	1.03±0.15	1.08±0.16	0.497	−
D360	1.05±0.17	1.05±0.20	1.02±0.15	1.10±0.15	0.402	−
Femoral neck BMD (g.cm^-2^)						
D21	0.81±0.12	0.82±0.12	0.80±0.12	0.84±0.12	0.363	−
D90	0.81±0.11	0.82±0.11	0.78±0.10	0.81±0.11	0.376	−
D180	0.80±0.11	0.82±0.12	0.78±0.09	0.82±0.11	0.247	−
D360	0.80±0.11	0.80±0.11	0.76±0.10	0.82±0.11	0.188	−

Abbreviations: *i-*groups, intervention groups; *re*, moderate-resistance-moderate-endurance; *Re*, high-Resistance-moderate-endurance; *rE*, moderate-resistance-high-Endurance; *C*, control; M, males; F, females; Non-CP, non-compliant; CP, compliant; hs, high-sensitivity; BMC, bone mineral content; BMD, bone mineral density.

**Fig 2:**
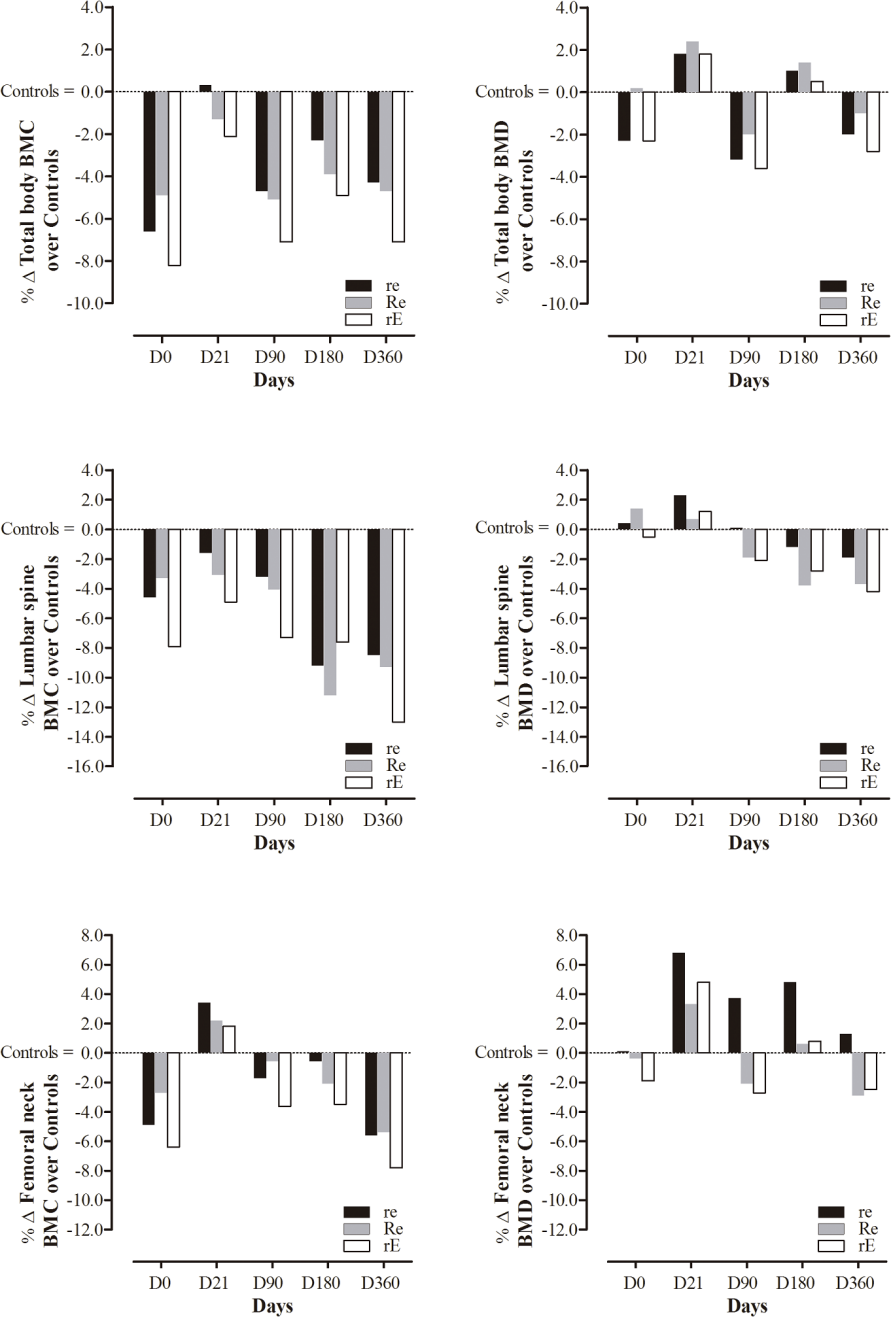
Changes (360 days) on total body bone mineral content (BMC) and density (BMD), lumbar spine BMC and BMD and femoral neck BMC and BMD for *re*, *Re* and *rE* groups. *re*: moderate-resistance-moderate-endurance; *Re*: high-Resistance-moderate-endurance; *rE*: moderate-resistance-high-Endurance. There were no significant differences in BMD and BMC parameters between *re*, *Re* and *rE* participants across the intervention. Participants in the intervention did not have significantly greater or lower bone mass or density development than controls.

There were no significant differences among MetS groups for BMC and BMD variables throughout the duration of the study (Table 2). Also, no MetS group differed in bone mass and bone density from controls (Fig 2).

### Cross-sectional effects of compliance

Compliance scores did not differ between groups. There was no difference between the diet compliance score and the physical activity compliance score. Overall, compliance score was 71.7±7.2% in compliant participants and 34.4±15.2% in non-compliant participants. This translates to an estimated 14.3±1.4 hours of physical activity in compliant participants and 6.8±3.0 in non-compliant participants.

**Fig 3:**
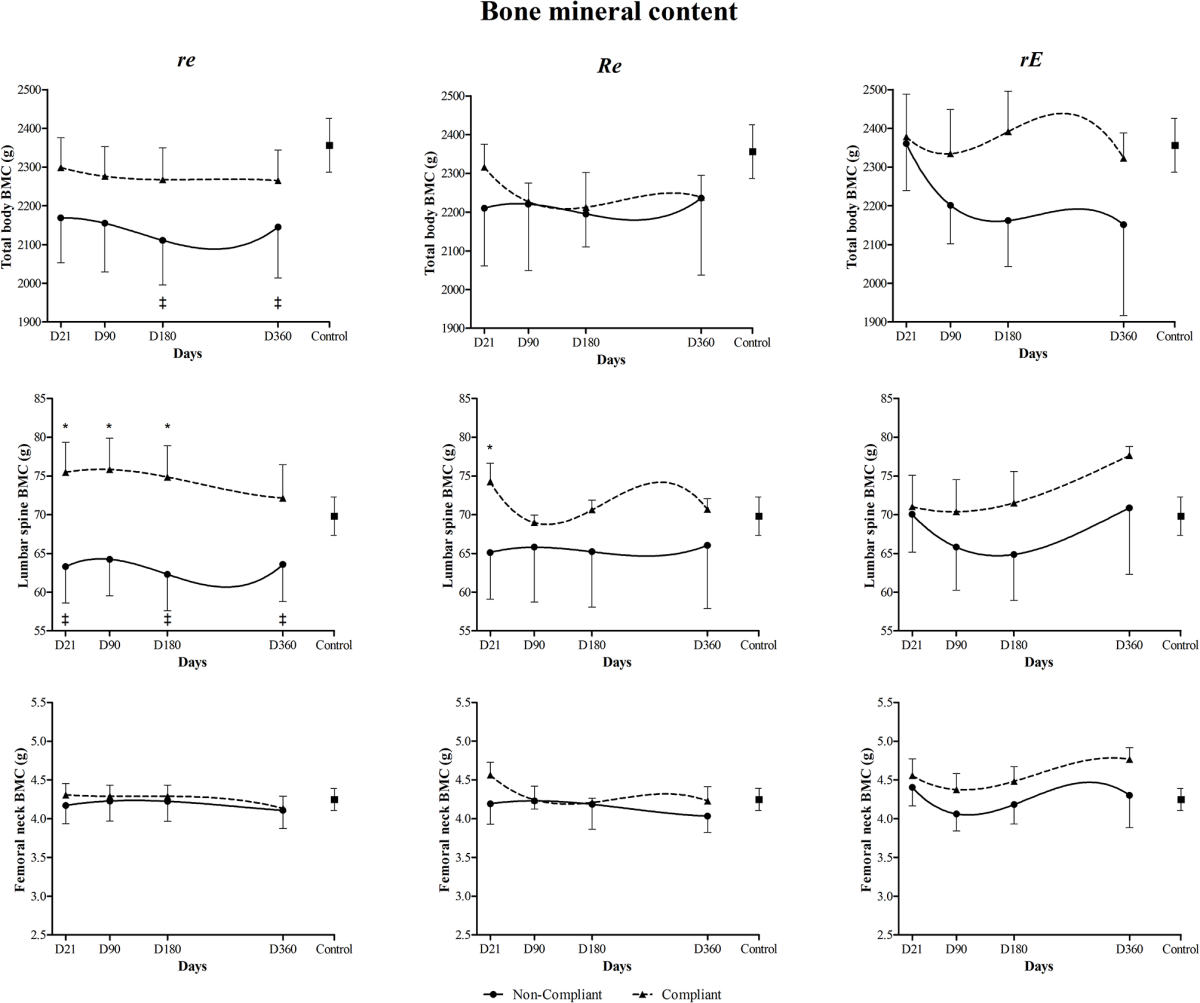
Compliance effect (360 days) on total body bone mineral content (BMC), lumbar spine BMC and femoral neck BMC for *re*, *Re* and *rE* groups. *re*: moderate-resistance-moderate-endurance; *Re*: high-Resistance-moderate-endurance; *rE*: moderate-resistance-high-Endurance. * Compliant participants significantly different from non-compliants (p<0.05). ‡ Non-compliant participants significantly different from controls.

**Fig 4:**
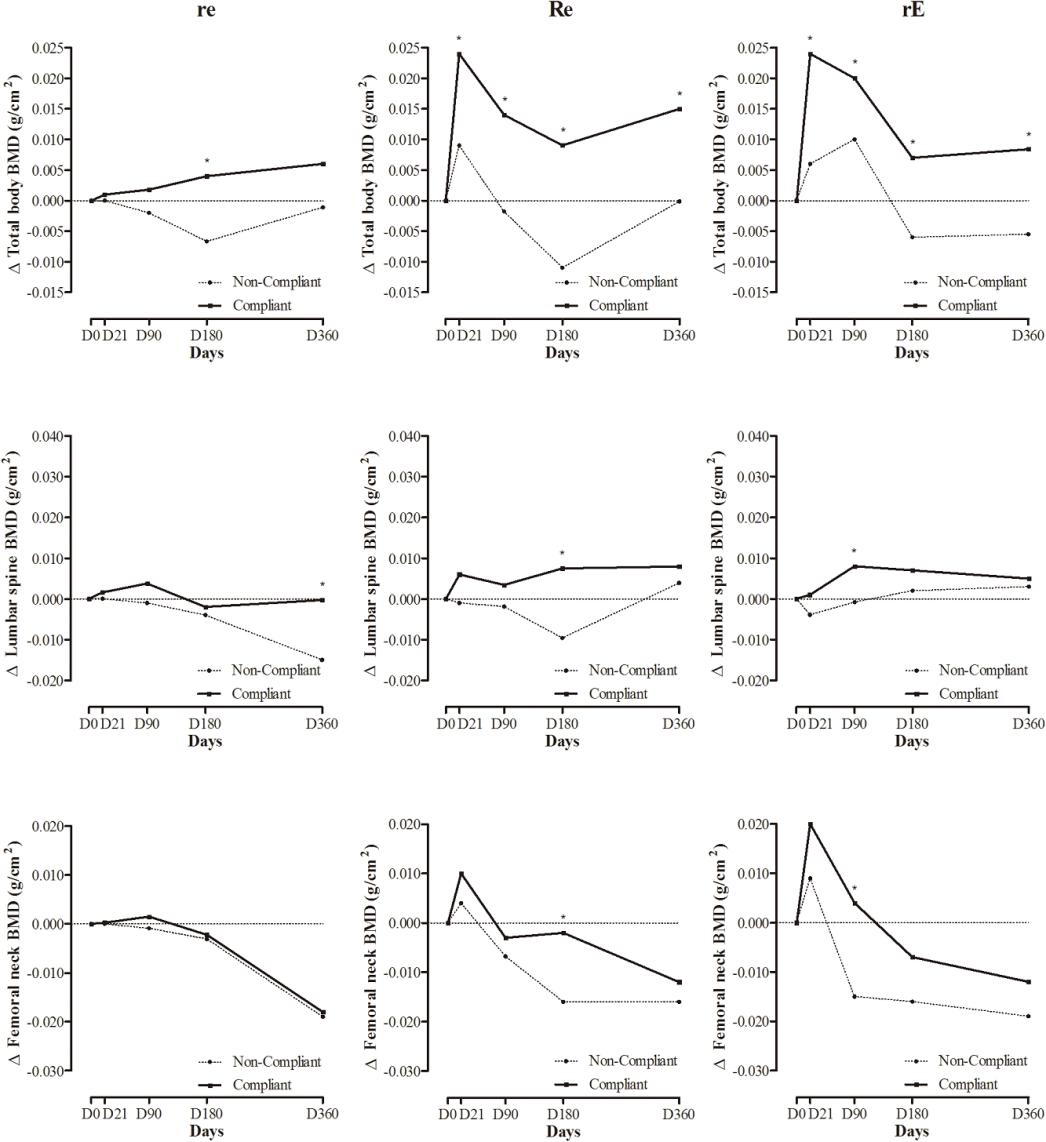
Compliance effect (360 days) on total body bone mineral density (BMD), lumbar spine BMD and femoral neck BMD for *re*, *Re* and *rE* groups. *re*: moderate-resistance-moderate-endurance; *Re*: high-Resistance-moderate-endurance; *rE*: moderate-resistance-high-Endurance. *Compliant participants significantly different from non-compliants (p<0.05).

Cross-sectional analyses of the compliance effects on BMC and BMD from D21 to D360 are displayed in Figs 3 and 4, respectively. At D21, compliant *re* and *Re* participants had significantly more lumbar spine BMC than their less-compliant *re* and *Re* peers. Similar findings were observed in compliant *Re* participants for lumbar spine BMD. Non-compliant *re* participants also had lower lumbar spine BMC than controls. At D90 and D360, compliant re participants maintained significantly higher lumbar spine BMC than non-compliant re participants. At D180 and D360, total body BMC and lumbar spine BMC were significantly lower in non-compliant *re* participants than in controls. At the end of the intervention program, compliant *rE* participants had greater femoral neck BMD than compliant *Re* participants and controls.

### Longitudinal multilevel modeling analyses

Tables 3 and 4 summarize the results from multilevel models for BMC and BMD at the total body, lumbar spine, and femoral neck. Total body BMC and BMD, lumbar spine BMC and BMD, and femoral neck BMD changed significantly over baseline within individuals at each measurement occasion. However, the three different modalities of exercise did not produce significantly different effects on any BMC or BMD measurements. The significant contribution of sex observed in five multilevel models indicates that female participants, regardless of the MetS group, had significantly less total body BMC (-556.45±60.82 g), lumbar spine BMC (-12.01±3.04 g) femoral neck BMC (-0.70±0.14 g), total body BMD (0.06±0.02 g.cm^-2^) and femoral neck BMD (0.04±0.02 g.cm^-2^) than their respective male counterparts when other confounders were controlled. An independent and significant increment of 8.14±3.27 g, 0.06±0.03 g.cm^-2^, and 0.04±0.02 g.cm^-2^ was also estimated for lumbar spine BMC, lumbar spine BMD, and femoral neck BMD, respectively, for those participants who were compliant with the exercise prescription throughout the intervention.

**Table 3:** Multilevel regression analysis of total body, lumber spine and femoral neck bone mineral content (BMC) aligned by days from study entry.

	BMC						
	Total body		Lumbar spine			Femoral neck	
Fixed explanatory variables	Estimates		Estimates			Estimates	
Constant	2073.2730±43.1213		69.3464±3.0431			3.8773±0.1015	
Days from start	-0.2123±0.0654		0.0019±0.0030			0.0002±0.0002	
Days from start ^2^	0.0004±0.0002		-0.000005±0.000008			-0.000001±0.0000006	
Age at study start	NS		NS			NS	
re *vs* Re	NS		NS			NS	
re *vs* rE	NS		NS			NS	
Female *vs* male	556.4508±60.8164		12.0111±3.0409			0.6962±0.1360	
Non-compliant *vs* compliant	NS		8.1360±3.2744			NS	
Changes in total body lean mass	-0.0068± 0.0019		-0.0003± 0.0001			NS	
Changes in total body fat mass	0.0030±0.0012		NS			NS	
Changes in central fat mass	NS		NS			NS	
Energy intake	-0.0044±0.0020		-0.0002±0.0001			NS	
Protein	NS		NS			NS	
Calcium	NS		NS			0.0001±0.00005	
Vitamin D	NS		0.0020±0.0009			0.0019±0.0009	
C-reactive protein	NS		NS			NS	
high-sensitivity C-reactive protein	NS		NS			NS	
Leptin	-0.0004±0.0002		NS			NS	
Variance-covariance matrix of random variables	Constant	Days from start	Constant	Days from start		Constant	Days from start
*Level 1 (within individuals)*							
Constant	1276.48±114.44		3.48±0.31			0.0307±0.0652	
*Level 2 (between individuals)*							
Constant	85896.77±12885.53		200.23±30.06			0.4258±0.0652	
Days from start centered	-8.65±4.59	0.0022±0.0032	0.0078±0.0143		0.00004 ± 0.00001	-0.00007±0.00004	0.00000002±0.00000007

Abbreviations: *re*, moderate-resistance-moderate-endurance; *Re*, high-Resistance-moderate-endurance; *rE*, moderate-resistance-high-Endurance.

Fixed effect values are presented as estimated mean coefficients ± SEE (standard error of estimate) of BMC in grams. Random effects values presented as estimated mean variance ± SEE (BMC) in grams^2^. Days from start was centered around 180 days. Changes in total body lean mass, total body fat mass and central fat mass (g) from study entry.

**Table 4:** Multilevel regression analysis of total body, lumber spine and femoral neck bone mineral density (BMD) aligned by days from baseline.

	BMD					
	Total body		Lumbar spine		Femoral neck	
Fixed explanatory variables	Estimates		Estimates		Estimates	
Constant	1.1119±0.0138		1.0956±0.0319		1.0727±0.1260	
Days from start	-0.000015±0.000006		-0.000005±0.0000011		-0.000047±0.000008	
Days from start ^2^	NS		NS		NS	
Age at study start	NS		NS		-0.0041± 0.000209	
re *vs* Re	NS		NS		NS	
re *vs* rE	NS		NS		NS	
Female *vs* male	0.0608±0.0201		NS		0.0440±0.0215	
Non-compliant *vs* compliant	NS		0.0618±0.0280		0.0415±0.0203	
Changes in total body lean mass	NS		NS		NS	
Changes in total body fat mass	- 0.0000011±0.0000003		NS		NS	
Changes in central fat mass	NS		NS		NS	
Energy intake	NS		NS		NS	
Protein	NS		NS		NS	
Calcium	NS		NS		NS	
Vitamin D	0.00014±0.00005		0.00022±0.00010		NS	
C-reactive protein	NS		NS		NS	
High-sensitivity C-reactive protein	NS		NS		NS	
Leptin	-0.0000002±0.0000001		NS		NS	
Variance-covariance matrix of random variables	Constant	Days from start	Constant	Days from start	Constant	Days from start
*Level 1 (within individuals)*						
Constant	0.00024±0.00001		0.00035±0.00003		0.00039±0.00004	
*Level 2 (between individuals)*						
Constant	0.00875±0.00131		0.02723± 0.00408		0.01131±0.00171	
Days from start centered	0.000000± 0.000000	0.000000± 0.000000	0.000000±0.000002	0.000000±0.000000	-0.000002± 0.000001	0.0000000002±0.0000000009

Abbreviations: *re*, moderate-resistance-moderate-endurance; *Re*, high-Resistance-moderate-endurance; *rE*, moderate-resistance-high-Endurance.

Fixed effect values are presented as estimated mean coefficients ± SEE (standard error of estimate) of BMD in g.cm^-2^. Random effects values presented as estimated mean variance ± SEE (BMD in g.cm^-2^)^2^. Days from start was centered around 180 days. Changes in total body lean mass, total body fat mass and central fat mass (g) from study entry.

Once the longitudinal changes in anthropometry, biochemistry, and nutrition were accounted for, total body BMC was observed to be negatively affected by the reduction of total body lean mass, negative energy balance, and decreased leptin. Additionally, decreases in total body fat mass contributed positively to total body BMC. Also, higher vitamin D significantly and positively contributed to total body BMD. Decreases in total body lean mass and negative energy balance significantly and independently contributed to decreases in lumbar spine BMC. In contrast, vitamin D intake had a positive association with lumbar spine BMC and BMD. Calcium intake and vitamin D were observed to be independent and significant predictors of femoral neck BMC. In contrast, other nutrition factors did not significantly contribute to observed variability within the changes in femoral neck BMD once age at baseline, sex, and compliance were accounted for.

## Discussion

The major finding of the present study was that despite minimal effects of exercise modalities, compliance with an intensive exercise program resulted in a significantly higher bone mass during energy restriction than non-compliance. More specifically, when assessed cross-sectionally, the effects of compliance on BMC and BMD was only noted during sporadic time points; however to draw conclusions based on these cross sectional observations, we ignored nutrition and other potential links to bone mass such as age, sex, anthropometry and compliance. When BMC and BMD parameters were assessed longitudinally and the independent effects of size, body composition, dietary intake and physical activity were accounted and individuals were allowed to have their own regression lines with separate intercepts and slope coefficients, an independent and significant increment of about 12%, 6% and 5% was noted for lumbar spine BMC, lumbar spine BMD and femoral neck BMD, respectively, for participants compliant with the exercise prescription across the intervention, whereas no changes were observed in less compliant participants.

### Effects of intervention and training modalities

Despite resistance training previously demonstrating increases in BMD at hip and lumbar spine sites [31], the results of the current study did not demonstrate differences in bone parameters related to modalities of exercise. The high volume of prescribed resistance training in all groups of physical activity (4 times a week i.e. 6 hours per week in our study) may explain the absence of differences in the results. In the present study, despite a rapid and sustainable weight loss in MetS participants, the BMC and BMD did not differ from controls. Despite that the amount of weight loss observed in participants in the present study had previously been associated with decreases in BMD and BMC [32,33,34], our data support that energy restriction did not necessarily result in bone loss [4,5,6,7]. The dose-response effect of exercise on bone is well established [35] and may explain this absence of bone loss, as demonstrated in comparisons between compliant and non-compliant participants.

### Effects of compliance

In our study, compliant participants increased BMC and BMD more than non-compliant participants, independent of the training groups. Thus, compliance appeared to be the most predominant factor for maximizing bone benefits [22]. Factors affecting compliance are numerous and should be taken into account in lifestyle interventions [36]. We report that all sites (total body, lumbar spine and femoral neck, for both BMC and BMD) in compliant participants benefited from the training, whereas previous literature has commonly reported site-specific improvements [37,38]. This could be due to the high level of physical activity (15–20 hours/week) imposed on individuals who were sedentary at baseline, with resistance training stimulating all parts of the body. However, it also demonstrates that our relatively high-volume training had no catabolic effect on bone metabolism, as encountered in overtraining [39]. Moreover, some bone parameters improved as acutely at the end of the residential program which reinforces the immediate potential benefits of physical activity [7,19,20]. As the resorption phase of bone modelling lasts 2–3 weeks and the formation phase 2–3 months [40], it would be surprising to have an increase in BMD and BMC after only three weeks of physical activity. However, such data have been previously reported on individuals with MetS [41]. Moreover, it has been shown that individuals with fractures may achieve a significant increase in BMC and BMD after three weeks [42].

### Other explaining variables

In the current study, caloric restriction decreased BMC, in agreement with previous studies [2,3]. We confirmed the importance of adequate sources of calcium [43,44] and vitamin D [45] on BMC and BMD with a multilevel approach. Then, we demonstrated that lean body mass, fat mass and leptin contributed to the prediction of changes in bone parameters. Fat tissue is an active endocrine organ secreting a host of molecules called adipokines, such as leptin. We have demonstrated the likelihood of bone-adiposity cross-talk in a recent literature review [46]. We were unable to report that insulin, pro-inflammatory cytokines, adiponectin, PAI-1, nor even bone metabolism markers were predictor variables of weight loss. Even if these factors have previously been linked to BMC and BMD [43], other studies have not taken into account repeated measurements, variations in the correlations over time, and the time difference between measurements. Nevertheless, throughout the one-year intervention, females showed fewer BMC and BMD benefits from physical activity than males. Even if being a male has a positive effect on BMD [44], we report more extensive monitoring of gender differences in BMC and BMD changes throughout a long-term intervention program than previously published.

### Limitations

This study has some limitations. A MetS group without any intervention or physical activity could have provided opportunities to distinguish the effects of a total intervention or physical activity from the diet. Implementing our intervention into health practices could be costly and our high volume training protocol (15–20 hours per week) may be difficult to comply with under non-supervised, community-based circumstances. However, we demonstrated the feasibility of such training volumes in a large sample size of sedentary obese individuals over 50 years old. The absence of direct bone metabolism measurements could also be seen as a limitation. Bone geometric data was not included in the current study. Eventually, despite some non-significant results, the multilevel hierarchical approach highlighted the effects of physical activity compliance on the prevention of bone loss classically associated with weight loss. However, compliance could only be judged *at posteriori* because a randomization based on compliance lacks predictability. Further studies should focus on psychological factors aimed at improving compliance in such long-term community-based intervention.

## Conclusion

Changes in bone parameters did not relate to the modalities of exercise. However, independent of physical activity groups, compliance with an intensive exercise program resulted in a significantly higher bone mass during energy restriction than non-compliance. Exercise is therefore beneficial to bone in the context of a weight loss program. Further studies should focus on maximising compliance in such long-term community-based interventions.

## Supporting Information

S1 CONSORT CONSORT guidelines. (DOC)

S1 Database Titles of columns are written without abbreviations. 999 = missing data.
(XLSX)

S1 Protocol Study protocol. (DOCX)
