# Correction: Multilevel Approach of a 1-Year Program of Dietary and Exercise Interventions on Bone Mineral Content and Density in Metabolic Syndrome - the RESOLVE Randomized Controlled Trial

**DOI:** 10.1371/journal.pone.0140307

**Published:** 2015-10-06

**Authors:** Daniel Courteix, João Valente-dos-Santos, Béatrice Ferry, Gérard Lac, Bruno Lesourd, Robert Chapier, Geraldine Naughton, Geoffroy Marceau, Manuel João Coelho-e-Silva, Agnès Vinet, Guillaume Walther, Philippe Obert, Frédéric Dutheil

Figs 2 and 4 are incorrect. The authors have provided corrected versions here.

**Fig 2:**
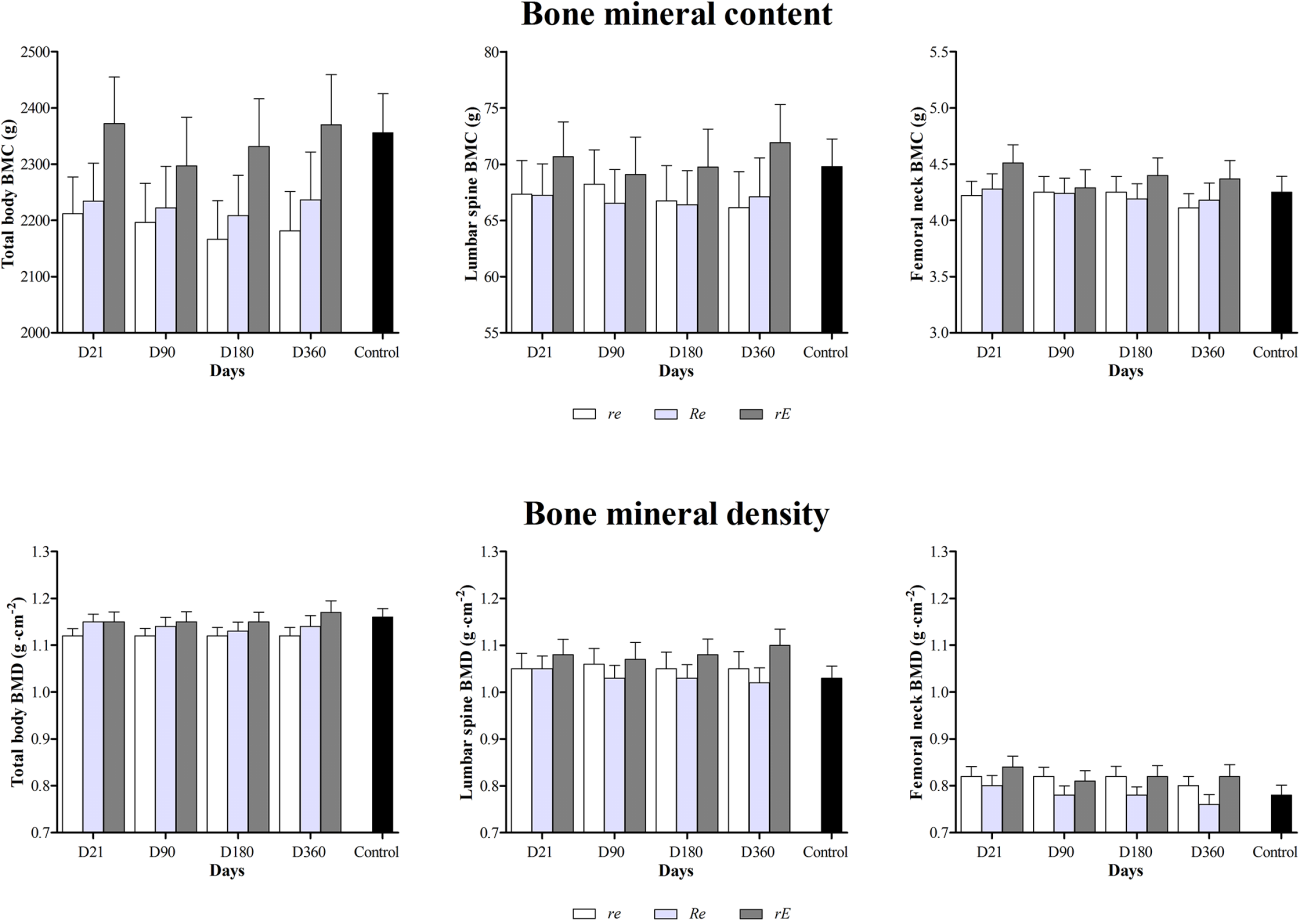
Changes (360 days) on total body bone mineral content (BMC) and density (BMD), lumbar spine BMC and BMD and femoral neck BMC and BMD for *re*, *Re* and *rE* groups. *re*: moderate-resistance-moderate-endurance; *Re*: high-Resistance-moderate-endurance; *rE*: moderate-resistance-high-Endurance. There were no significant differences in BMD and BMC parameters between *re*, *Re* and *rE* participants across the intervention. Participants in the intervention did not have significantly greater or lower bone mass or density development than controls.

**Fig 4:**
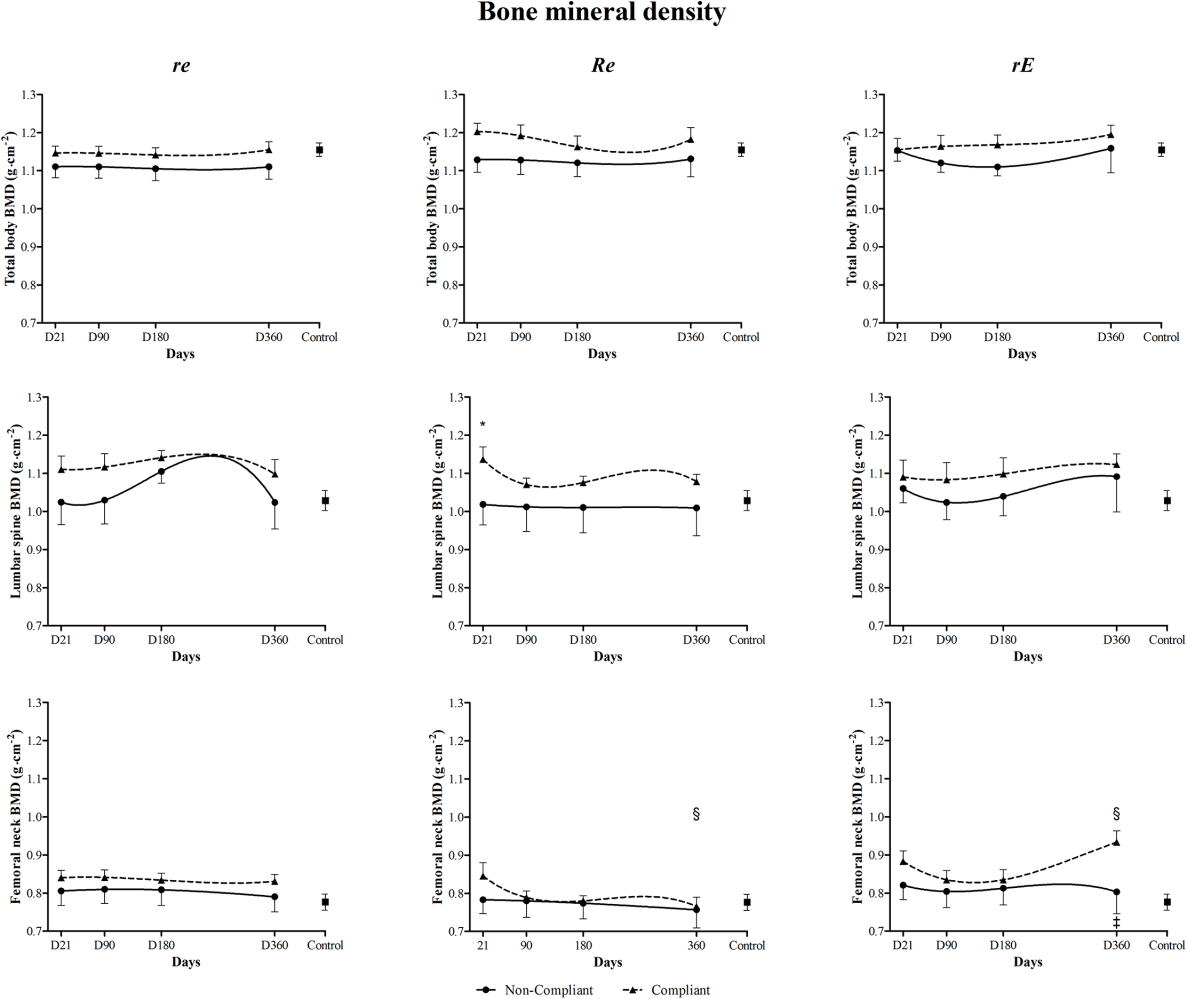
Compliance effect (360 days) on total body bone mineral density (BMD), lumbar spine BMD and femoral neck BMD for *re*, *Re* and *rE* groups. *re*: moderate-resistance-moderate-endurance; *Re*: high-Resistance-moderate-endurance; *rE*: moderate-resistance-high-Endurance. ^*^Compliant participants significantly different from non-compliants (p<0.05). ‡ Compliant participants significantly different from controls. § Significant difference *Re* compliants vs. *rE* compliants.
