# Supplementary material for: Multilevel Approach of a 1-Year Program of Dietary and Exercise Interventions on Bone Mineral Content and Density in Metabolic Syndrome – the RESOLVE Randomized Controlled Trial
Source: PLoS One. 2015 Sep 16;10(9):e0136491. doi: 10.1371/journal.pone.0136491 (PMC4574281; doi:10.1371/journal.pone.0136491)
Supplement: S1 Protocol — (DOCX) [file pone.0136491.s003.docx]

**Effect of Physical Activity and Diet on the Treatment of Metabolic Syndrome**

Verified January 2011 by University Hospital, Clermont-Ferrand

First Received on June 9, 2009.   Last Updated on January 18, 2011

| **Sponsor:** | University Hospital, Clermont-Ferrand |
| --- | --- |
| **Collaborator:** | Fondation Coeur et Artères |
| **Information provided by:** | University Hospital, Clermont-Ferrand |
| **ClinicalTrials.gov Identifier:** | NCT00917917 |


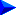
**Purpose**

Metabolic syndrome has been defined as a group of associated risk factors for cardio-vascular diseases and diabetes. It is usually treated with an association of restrictive diet, physical exercise and drugs. Nevertheless the type of exercise associated to reduction in cardio-vascular risks is not yet fully defined. Long term effects of such hygienic-diet programs are of great importance since it is well-known that compliance to such treatment are of short duration, namely when subjects return in routine life. Metabolic syndrome volunteer subjects (n=90), aged 50 to 70 yrs will be randomly assigned to 3 groups of investigation.One group will perform mostly resistance activity, a second mainly endurance activity and the third one will be composed of subjects not exercising a lot. All subjects will have the same restrictive diet (500-700 kcal/d) After the initial training (3 weeks), they will return home with diet and physical program advises (personal compliance). They will be followed for one year (at 3, 6 and 12 months) Such a design may allow to find out the type of activity and power that are the best to reduce metabolic syndrome parameters and cardio-vascular risk factors.

The primary outcome variable is the reduction in abdominal circumference, which is the main criteria of MS.

| [**Condition**](http://clinicaltrials.gov/ct2/help/conditions_desc) | [**Intervention**](http://clinicaltrials.gov/ct2/help/interventions_desc) |
| --- | --- |
| Metabolic Syndrome | Behavioral: Type of physical activity (resistance, endurance) Behavioral: Restrictive diet |

| Study Type: | Interventional |
| --- | --- |
| Study Design: | Allocation: Randomized Endpoint Classification: Efficacy Study Intervention Model: Parallel Assignment Masking: Open Label Primary Purpose: Prevention |
| Official Title: | Role of Combined Intervention of Physical Activity and Nutrition in Metabolic Syndrome Treatment on Cardio-vascular Risk and Muscular- Skeletal Functions in Human Subject. Analysis of Patient's Compliance in Patient's Follow-up. |

**Resource links provided by NLM:**

[MedlinePlus](http://www.nlm.nih.gov/medlineplus/) related topics: [Exercise and Physical Fitness](http://clinicaltrials.gov/ct2/bye/YQoPWw4lZX-i-iSxudhWudNzlXNiZip9m67PvQ7xzwhaLwS9zThHSBczF67Gu6o3lT1geBcGv6NzWdNHFwSxlihLv.) [Metabolic Syndrome](http://clinicaltrials.gov/ct2/bye/xQoPWw4lZX-i-iSxudhWudNzlXNiZip9m67PvQ7xzwhaLwS9m61PkBN9vQ7gFT7x5wN9m6DxlihLv.)

[U.S. FDA Resources](http://clinicaltrials.gov/ct2/info/fdalinks)

**Further study details as provided by University Hospital, Clermont-Ferrand:**

Primary Outcome Measures:

- Decreased abdominal fat mass, measured after one year. Such measured will be done repetitively at Day 0, 21 and at 3, 6 and 12 months using abdominal circumference and DXA Decreased cardio-vascular risks [ Time Frame: at Day 0, 21 and at 3, 6 and 12 months using abdominal circumference and DXA ] [ Designated as safety issue: Yes ]

Secondary Outcome Measures:

- All the following measures will be done at day 0, 21 and at month 3, 6 and 12. Level of physical activities of each types Physical performances, Food intake and equilibrium Other Metabolic Syndrome inc [ Time Frame: at day 0, 21 and at month 3, 6 and 12. ] [ Designated as safety issue: Yes ]

| Estimated Enrollment: | 120 |
| --- | --- |
| Study Start Date: | May 2009 |
| Estimated Study Completion Date: | October 2012 |
| Estimated Primary Completion Date: | October 2011 (Final data collection date for primary outcome measure) |

Intervention Details:

Behavioral: Type of physical activity (resistance, endurance)

To determine which type of physical activity is the best to reduce metabolic syndrome parameters especially abdominal circumference.

Group 1 will perform mostly resistance activities, group 2 mostly endurance activities, and Group 3 performing both activities at low level, serves as a control group for physical activities.

Behavioral: Restrictive diet

All subjects will have the same restrictive diet (500- 700 kcal/d).

**Detailed Description:**

The protocol is designed to determine which type of physical activity is the best to reduce metabolic syndrome parameters especially abdominal circumference.

90 Metabolic syndrome (MS) Patients will be recruited by advertising and checked for MS criteria. They will have a VO2max test in order to be sure they can perform physical activities safely. After being checked, patients will have an eight day period to think about participation and to ask questions before signing consent. They have to feel 3 questionnaires, one about regular physical activities, one about food intakes and a psychology one to measure reluctance to the program. After written informed consent obtained, patients will be randomly assigned to one of 3 groups of physical activity Group 1 will perform mostly resistance activities, group 2 mostly endurance activities, and group 3 performing both activities at low level, serves as a control group for physical activities and also to determine the importance of food reduction and food equilibrium in the treatment.

All subjects will have the same restrictive diet (500- 700 kcal/d). They will be followed for one year (at 3, 6 and 12 month), continuing at home the same program (diet and exercise training).

30 healthy subjects will be recruited for cross-sectional comparison (They will not follow any intervention, but will have the same investigation, only once).

Measured parameters Before and after the 3-week program and at 3, 6 and 12 months, the following measurements will be made Level of physical activities quantifying heart rate in each activity during the training 3 weeks and estimated thereafter on the same parameter.

Physical capacities With the 6 minute walking test, Food intake and equilibrium measured by full week records before training, during training and monthly thereafter. They will be quantified by a trained dietician using the reference French Cidal tables.

Metabolic syndrome factors Body composition including weight, height, abdominal circumference, total and torcular lean and total and abdominal fat mass measured by DXA Cardiac diastolic and systolic functions by means of standard, Tissue Doppler imaging and 2D-strain echocardiogram Vascular structure function in conduit and resistance arteries and microvascular reactivity Biological parameters with glycemic control : insulinemia and glaced haemoglobin Inflammatory syndrome and related cytokines: CRP, 1GPA, IL-6, TNF-, Il-12 and IL-10 blood protein: Albumine and transthyretin Appetite hormone: Leptine, adiponectin, Gremlin and CCK osteocalcin, BASP and CTx

Statical analysis Subject numbers were calculated from the results of a pilot. A statistical significance (p=0,05) may be reached with 22 subjects in group 1 and 2 for a difference of 0,6 kg of abdominal fat mass.

For cross-sectional comparison, healthy and MS subjects will be compared by unpaired Student test. The 3 groups of patients will be compared using a repeated measure ANOVA. If positive, a post-hoc test for mean comparison will be performed.

A correlation matrix will analyse relationships between studied parameters. A principal component analysis will allow to determine the reciprocal weight of positive explicating factors.

The study is done applying French and international regulations


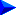
**Eligibility**

| Ages Eligible for Study: | 50 Years to 70 Years |
| --- | --- |
| Genders Eligible for Study: | Both |
| Accepts Healthy Volunteers: | Yes |

**Criteria**

Inclusion Criteria:

- 50-70 years old from both sexes
- with metabolic syndrome
- affiliated to a social security system
- able to practice maximal physical exercises based on VO2 max
- able to sign inform consent

Exclusion Criteria:

- recent (6 month) major health conditions and patients with recurrent health problems (1 per year)
- patients who are not capable to perform VO2 max without abnormalities
- dyserection treated patients
- patients with insufficient comprehensive ability to feel questionnaire and/or to change habits


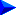
**Contacts and Locations**

Please refer to this study by its ClinicalTrials.gov identifier: NCT00917917

**Contacts**

| Contact: Patrick Lacarin | 04.73.75.11.95 | [placarin@chu-clermontferrand.fr](mailto:placarin%40chu-clermontferrand.fr?subject=NCT00917917,%20CHU-0053,%20Effect%20of%20Physical%20Activity%20and%20Diet%20on%20the%20Treatment%20of%20Metabolic%20Syndrome) |
| --- | --- | --- |

**Locations**

| **France** | |
| --- | --- |
| CHU Clermont-Ferrand | **Recruiting** |
| Clermont-Ferrand, France, 63003 | |
| Contact: Patrick Lacarin     04.73.75.11.95     [placarin@chu-clermontferrand.fr](mailto:placarin%40chu-clermontferrand.fr?subject=NCT00917917,%20CHU-0053,%20Effect%20of%20Physical%20Activity%20and%20Diet%20on%20the%20Treatment%20of%20Metabolic%20Syndrome) | |

**Sponsors and Collaborators**

University Hospital, Clermont-Ferrand

Fondation Coeur et Artères

**Investigators**

| Principal Investigator: | Bruno Lesourd, MD | University Hospital, Clermont-Ferrand |
| --- | --- | --- |


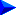
**More Information**

| Responsible Party: | Patrick Lacarin, CHU Clermont-Ferrand |
| --- | --- |
| ClinicalTrials.gov Identifier: | [NCT00917917](http://clinicaltrials.gov/ct2/show/NCT00917917) |
| Other Study ID Numbers: | CHU-0053 |
| Study First Received: | June 9, 2009 |
| Last Updated: | January 18, 2011 |
| Health Authority: | France: Ministry of Health |

Keywords provided by University Hospital, Clermont-Ferrand:

| Metabolic syndrome exercise training abdominal fat mass endothelial function diastolic function |
| --- |
|  |

Additional relevant MeSH terms:

| Metabolic Syndrome X Insulin Resistance Hyperinsulinism Glucose Metabolism Disorders Metabolic Diseases |
| --- |

ClinicalTrials.gov processed this record on August 01, 2012
